# A dataset of acoustics emissions recordings of woodboring insects in wood and cultural objects, context images and remarks

**DOI:** 10.1016/j.dib.2026.112461

**Published:** 2026-01-13

**Authors:** Tom Marti, Cécile Costa, Emmanuel de Salis, Laura Brambilla, Stefano Carrino

**Affiliations:** a HES-SO // University of Applied Sciences and Arts Western Switzerland, Haute Ecole Arc Ingénierie, Espace de l’Europe 11, 2000 Neuchatel, Switzerland; b HES-SO // University of Applied Sciences and Arts Western Switzerland, Haute Ecole Arc Conservation-restauration, Espace de l’Europe 11, 2000 Neuchatel, Switzerland

**Keywords:** Acoustic emission, Wood, Cultural heritage, Machine learning, Infestation, Time-series

## Abstract

This dataset presents acoustic emission (AE) recordings collected from woodboring insect-infested and non-infested wood samples and cultural heritage objects. Data acquisition was conducted across four institutions: Haute École Arc (HE-Arc), Switzerland; Canadian Museum of History (CMH), Canada; National Gallery of Canada (NGC), Canada; and Musée National de l'Automobile (MNA), France; from April to July 2025.

The recordings were captured using Vallen VS900-M sensors with AEP5 preamplifiers set to 34dB gain and AMSY-6 4-channel chassis, employing continuous acoustic emission monitoring at 2 MHz sampling rate. Each experiment utilized three sensors positioned on test objects and one reference sensor facing up to record ambient noise conditions. The dataset comprises approximately 440.9 hours of recordings distributed across the four collection sites.

The dataset includes four main components: raw Vallen AE database files (.tradb format), processed statistical data exported as CSV files, contextual images documenting setups and sensor placements, and Python script for statistical data processing. Each experiment is documented with duration, material specifications, coupling methods (renaissance wax, cyclododecane, or mechanical fastening), environmental conditions, and infestation labels.

The dataset's structure enables multiple research applications. The time-series statistical features and binary classification labels (infested/non-infested) provide a foundation for supervised machine learning model development. The diverse experimental conditions across four geographic locations, varying coupling methods, and different ambient environments offer opportunities to evaluate model generalization and robustness. Reference sensor recordings captured simultaneously with each experiment allow for ambient noise characterization studies and development of noise filtering methodologies. The combination of raw acoustic data and contextual documentation makes this dataset suitable for comparative studies of different signal processing approaches and feature extraction techniques in acoustic emission analysis for heritage conservation applications.

Specifications Table

**Table:** 

Subject	Computer Sciences
Specific subject area	Acoustics Emissions (AE) recordings of infested and not infested wood and cultural objects.
Type of data	- Raw Vallen AE Databases (.tradb files) - CSV of Computed Statistics on Raw files - Images of experiments - Scripts (Python)
Data collection	The dataset contains recordings of infested and not infested woods or cultural objects by woodboring insects. It includes a strong context description and an ambient noise recording. Sample have been recorded using a Vallen VS900-M as Sensor, a Vallen AEP5 set to 34dB as preamplifier and a Vallen AMSY-6, 4 channels as chassis. The sampling is 2MHz and continue. Each experiment is composed of 3 sensors disposed on wood and 1 near the wood with the sensible part facing up. A picture of the set-up is taken. At the end of the recording, duration, labels (defined by AE expert) and remarks are consigned into a csv file.
Data source location	• Haute École Arc (HE-Arc), Switzerland Latitude: 46.997638, Longitude: 6.938585; • Canadian Museum of History (CMH), Canada Latitude: 45.4298365, Longitude: -75.708575; • National Gallery of Canada (NGC), Canada Latitude: 45.4295263, Longitude: -75.6988125; • Musée National de l’Automobile, (MNA), France Latitude: 47.7650786, Longitude: 7.3396819.
Data accessibility	Repository name: Zenodo Data identification number: https://doi.org/10.5281/zenodo.16315620 [1] Direct URL to data: https://zenodo.org/records/16315621
Related research article	none

## 1 Value of the Data

• This dataset provides rich contextual information for each experiment. This comprehensive documentation allows researchers to properly interpret acoustic emissions signals, account for environmental and coupling effects, and facilitate reuse and comparative studies without redundant experiments.
• We make a point to record experiments in as many different ambiences as possible and try to record the ambiance background noise. To achieve this, the experiments were carried out in 4 different locations, with the aim of creating a variety of activities around them. The time of the recording and the activity around them is variable too, for example by night, by day, with truck loading around, outside of the building, etc. To record the ambiance of each location during each experiment, a sensor has been set to point at the sky and used to record the ambient noise.
• Data is easy to transform into a standard format for machine learning. The script “tradv2csv.py” transforms the raw Vallen databases to CSVs and aggregates references sensors with the signal sensors. Each hit (a recording of 1′024 samples) is decomposed into a few statistics.
• The dataset is intended to be used as time series to feed machine learning or deep learning models. Converting the data into time series of statistical features makes it well-suited for these applications.
• Alternatively, the data can be used to analyze the influence of ambient noise on acoustics emissions or explore noise reduction in preprocessing.

## 2 Background

Insect infestation represents an important source of degradation in museum collections and is a major challenge to detect and treat [2,3]. In the case of woodboring insects, it is common to confuse active infestation with traces of old ones [2].

The most used detection techniques are observation of infestation signs and quarantine, who are time-consuming and offer no guarantee [2,4]. Sticky traps are also widely used but fail to pinpoint precisely which object is affected within a collection [2,3].

Acoustic emission (AE) offers a promising and non-invasive approach for determining the condition of heritage collections [5]. When coupled with deep learning, it can provide an automated way to determine the state of collection without the need of an AE expert [[6], [7], [8], [9], [10]].

This combination would reduce the need for anoxia and thermal treatment, which are costly and sometimes incompatible with certain materials or objects. Museums would benefit in terms of precision, resources, and time [2,3].

The current lack of public datasets about acoustics emissions on this topic forced us to create an annotated dataset before starting to explore the different ways to achieve our goal.

## 3 Data Description

This dataset is distributed in four zipped archives, one CSV description file, and an experiment base for reproduction. “raw.zip” and “csv.zip” contain respectively the raw data (tradb database files from Vallen software) and derived statistical data (CSV files containing statistics computed from the tradb files) as time series. The script archive contains the script to compute the statistics and scripts to understand data. The “images.zip” file contains the reference images of each experiment. “experiment_description.csv” file links the different parts of the dataset. The “source.pridb” file is a base to reproduce the experiment with the same settings. The “README.md” file describes how to merge the raw archive.

The raw archive contains around 440.9 hours of recordings distributed across 4 sites. Each experiment is stored in a database file (.tradb file), an SQLite database you can explore using an SQLite database explorer.

A database file is composed of hits. Each hit is a recording of 1′024 samples collected at 2 MHz. Hits are equally spaced in time (1 every 0.04096s). Each entry on database is 1 hit for a sensor. A database is composed of four tables. Table 1, Table 2–3.

**Table 1:** Database tables and their descriptions.

Table	Description
tr_data	Each tr_data row is one hit of 1 sensor: SetID tags it, Time, TRAI and Status record when and how it fired, ParamID and Chan link to acquisition settings and channel, Pretrigger, Thr, SampleRate, Samples and DataFormat describe its waveform capture, and Data holds the raw samples.
tr_fieldinfo	The tr_fieldinfo table maps each data field name to its measurement unit and its descriptive parameter label.
tr_globalinfo	Each tr_globalinfo row stores a unique Key and its corresponding Value, defining global parameters or settings for the dataset.
tr_params	Each tr_params row defines channel parameters: ID is the primary key, SetupID links to the experimental setup, Chan identifies the channel, ADC_µV gives the ADC conversion factor in microvolts, and TR_mV specifies the trigger level in millivolts.

**Table 2:** Experiment description file column explanation.

Column	Description
name	Name of the experiment, name of the “tradb” file and name of the main image.
image	Global experiment image name.
precision	List of other images related to the experiment.
duration	Duration of the experiment in minute.
material	Material tested, currently always wood.
coupling	Medium between the sensor and the objects, mostly cyclododecane or renaissance wax.
wet	If the wood is dry or wet/humid.
chan 1	Label of channel 1. Reference (ref), infested or not infested.
chan 2	Label of channel 2. Reference (ref), infested or not infested.
chan 3	Label of channel 3. Reference (ref), infested or not infested.
chan 4	Label of channel 4. Reference (ref), infested or not infested.
remarks	Remarks such as ambiance, coupling details, infestation status details, duration details.

**Table 3:** Description of statistics of csv files.

Statistics name	Description
(ref_)sum	Total of all signal values
(ref_)mean	Arithmetic average of the signal
(ref_)std	Standard deviation around the mean
(ref_)median	Middle value of sorted signal
(ref_)min	Smallest signal value
(ref_)max	Largest signal value
(ref_)q1	25th percentile
(ref_)q2	50th percentile (median)
(ref_)q3	75th percentile
(ref_)fft_max	Maximum magnitude in the frequency spectrum
(ref_)fft_mean	Average spectral magnitude
(ref_)fft_std	Standard deviation of spectral magnitudes
(ref_)fft_peak_freq	Frequency with highest amplitude
(ref_)skewness	Measure of distribution asymmetry
(ref_)kurtosis	Measure of distribution “tailedness”
(ref_)entropy	Shannon entropy of absolute values
(ref_)zcr	Number of zero crossings
(ref_)energy	Sum of squared signal values (total power)

Each experiment has a database, and one or more images linked, except for experiment HEARC_007 where images are missing. Images show the placement of sensors on objects or samples. These images are informative, with each filename providing details about the content shown.

*_number* indicates that the image displays a specific sensor rather than the complete object.

*_c(number)* indicates a copy of an image or a different angle.

For example, the image “CMH_003_3_c2.png” breaks down as: CMH_003 = the object identifier; 3 = focuses on sensor 3; c2 = the second picture/angle of this sensor. The physical notes directly provided in images showing an experiment name should be ignored as they are not reliable.

The CSV file “experiment_description.csv” describes each experiment. It makes the link between “.tradb” files, images, labels and remarks. It contains 12 columns.

Each experiment includes a reference sensor facing the sky to record ambient conditions. This sensor is marked with 'ref' in the appropriate channel column (chan 1-4) to indicate which channel contains the reference measurements. Fig. 1, Fig. 2, Fig. 3, Fig. 4, Fig. 5, Fig. 6, Fig. 7, Fig. 8, Fig. 9–10.

**Fig. 1:**
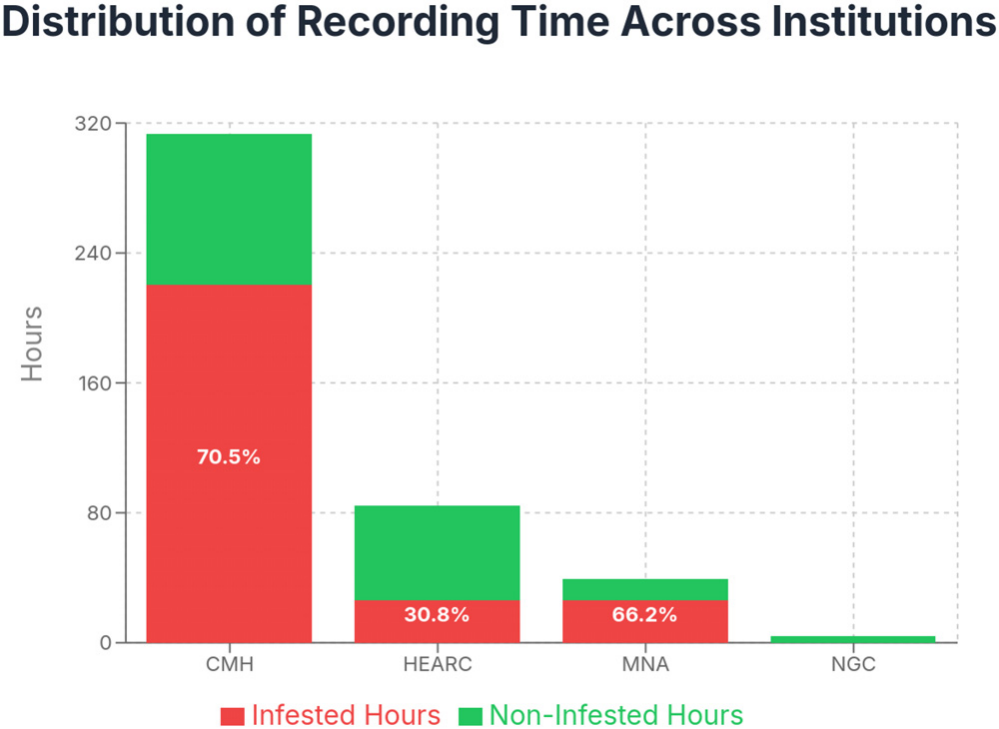
Stacked bar chart showing the total recording hours available from each institution, with infested periods (red) and non-infested periods (green). Percentages indicate the proportion of infested time for each institution.

**Fig. 2:**
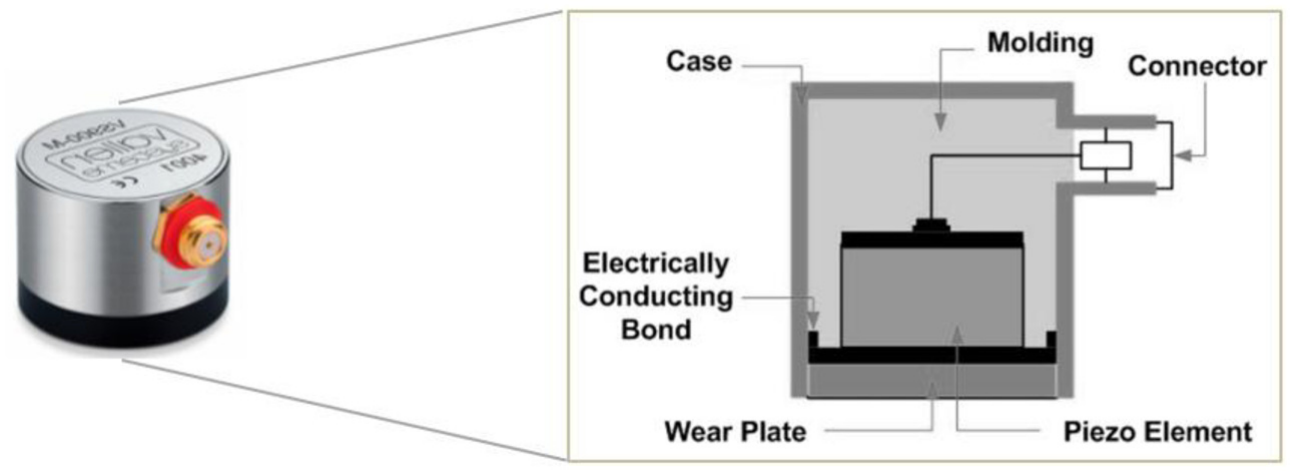
Composition of an AE sensor (©Juan Luis Ferrando Chacon).

**Fig. 3:**
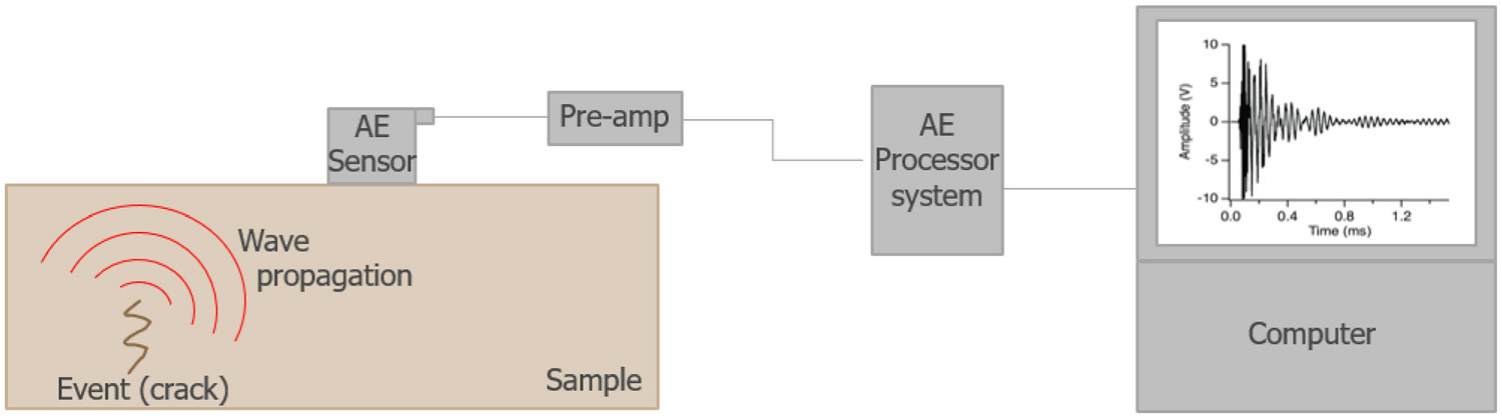
Simplified diagram of the AE acquisition chain.

**Fig. 4:**
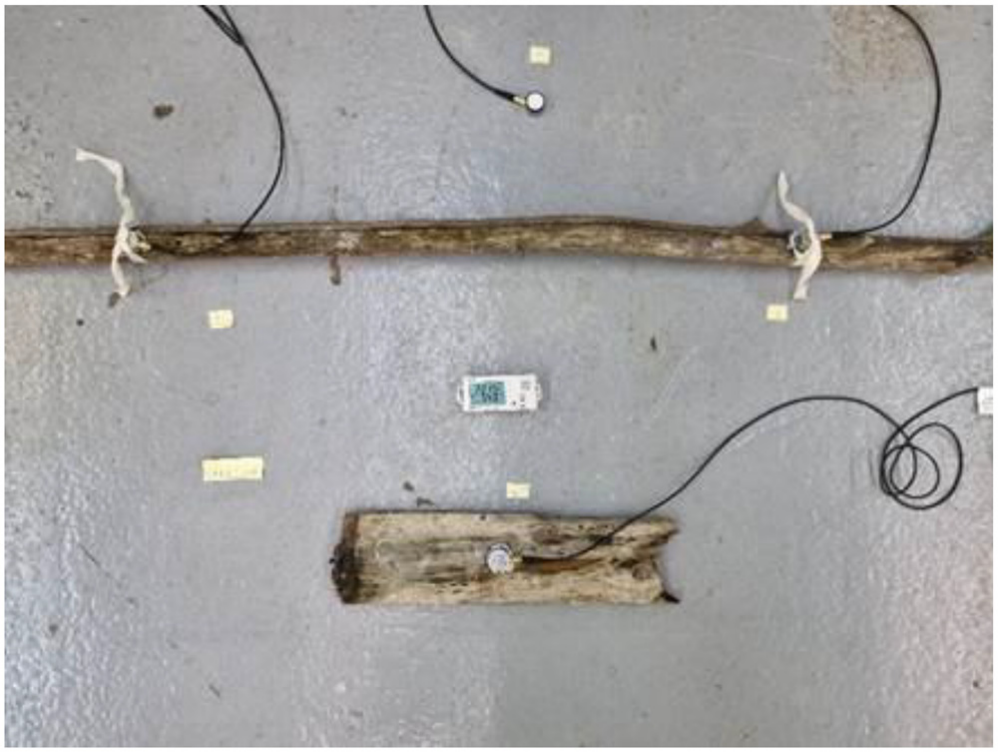
Example of a typical setup.

**Fig. 5:**
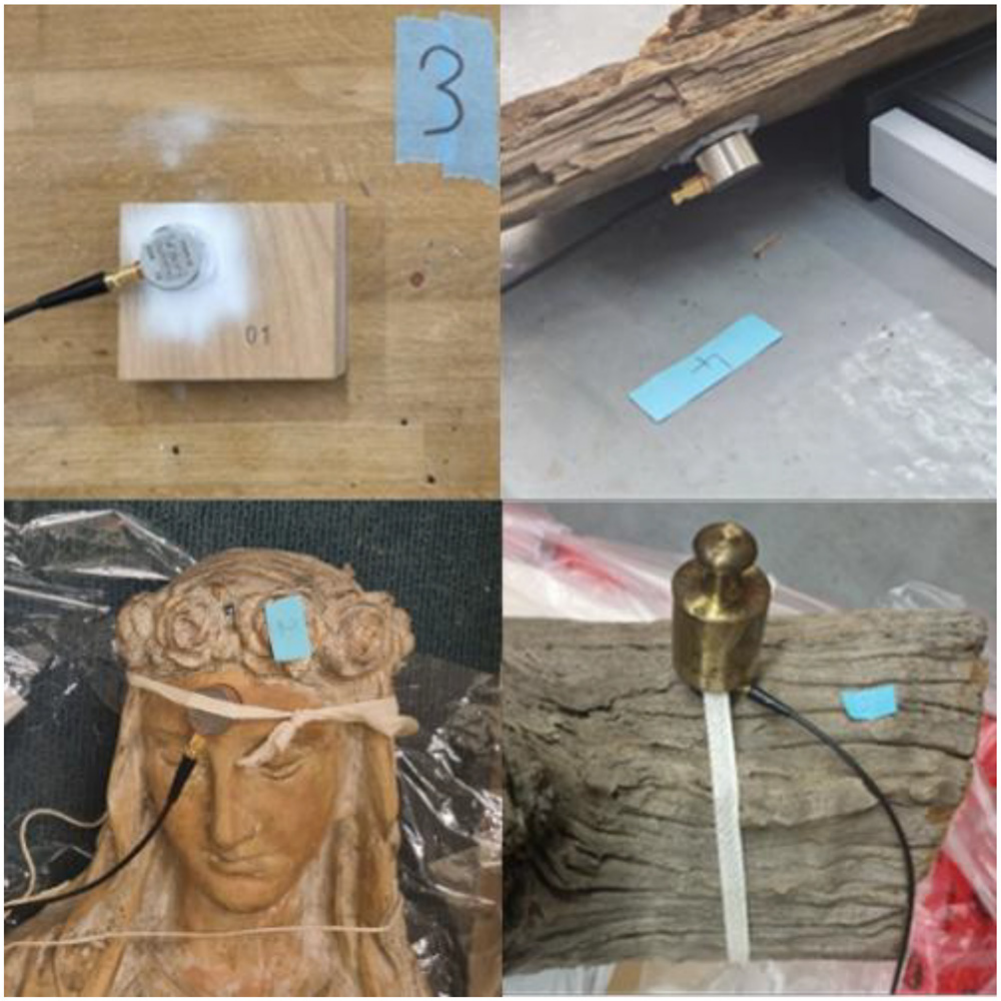
Top left: Sensor fixed with cyclododecane; Top right: Sensor fixed with renaissance wax; Bottom left: Sensor fixed only with ribbons; Bottom right: Sensor fixed with ribbons and weight.

**Fig. 6:**
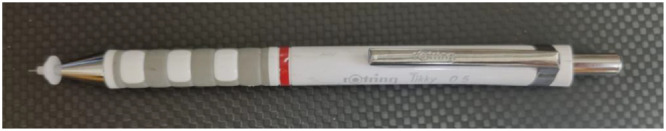
Hsu-Nielsen Source (mechanical pencil equipped with breaking angle guide).

**Fig. 7:**
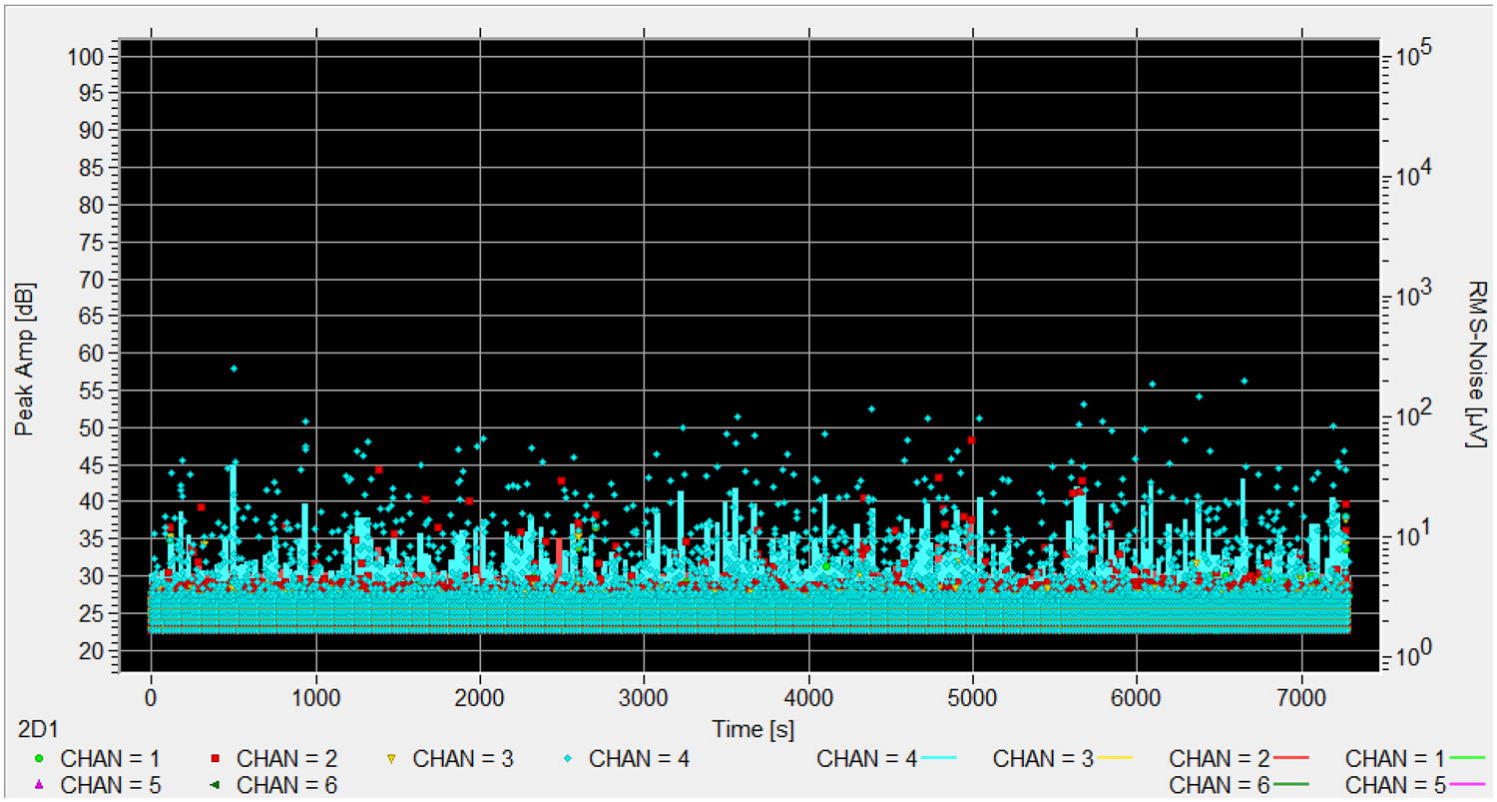
Hits amplitude in time of experiment MNA_002, the channel 2 (red) and 4 (blue) are infested.

**Fig. 8:**
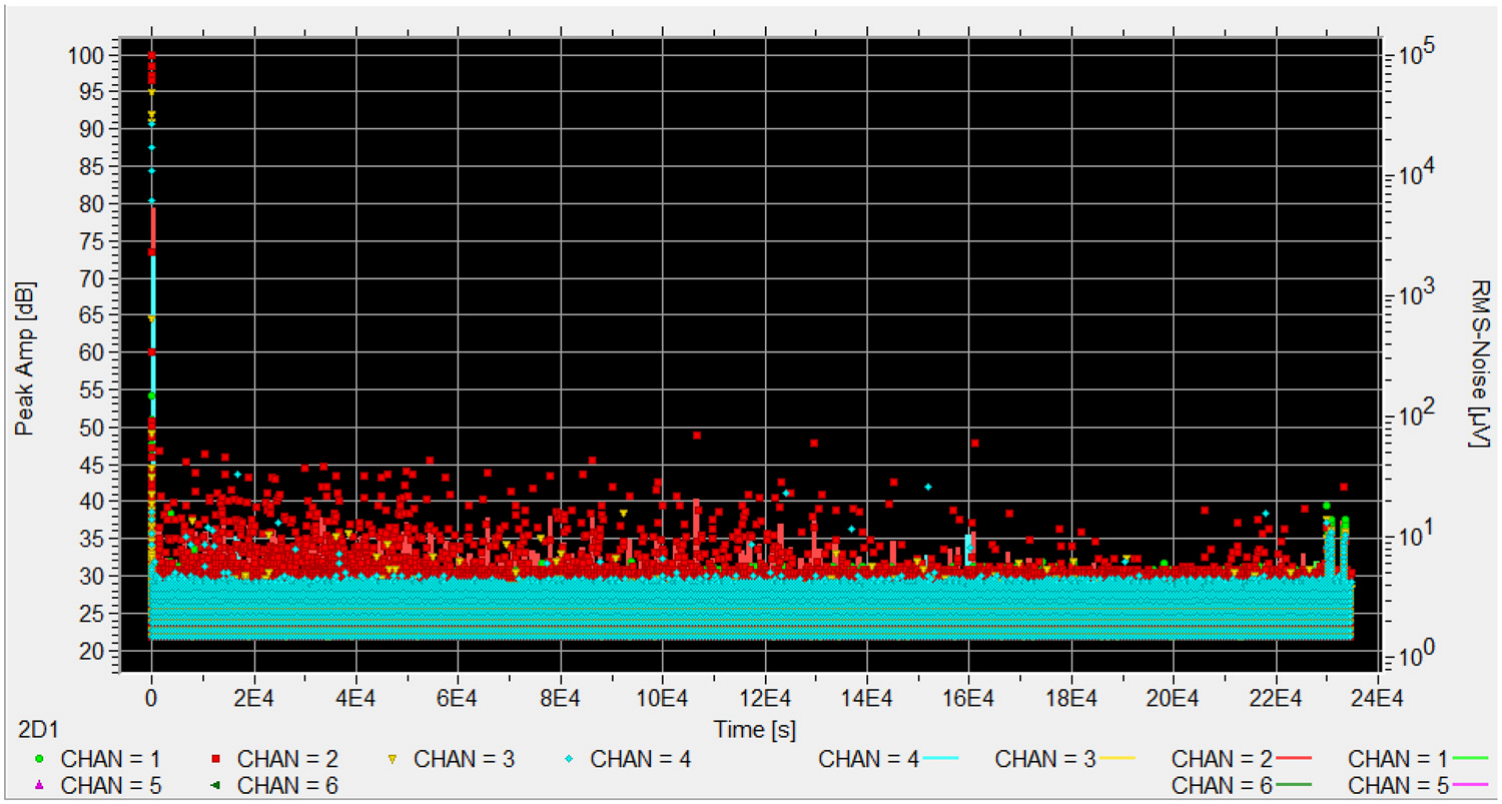
Test CMH_B_N_034 showing signs of infestation on CHAN2 before freeze-kill protocole.

**Fig. 9:**
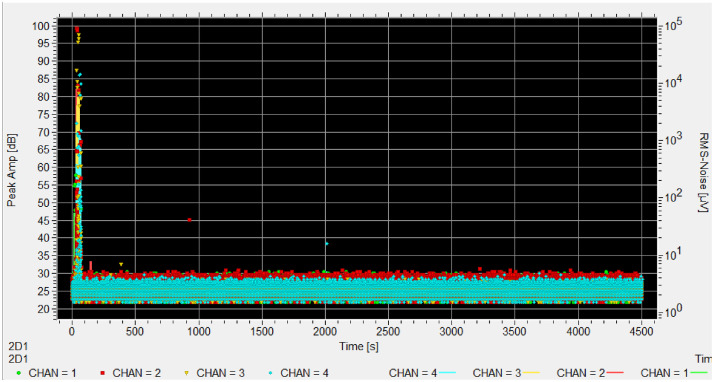
Test CMH_B_N_035 (same wood sample) after freeze-kill protocole. No more signs of infestation.

**Fig. 10:**
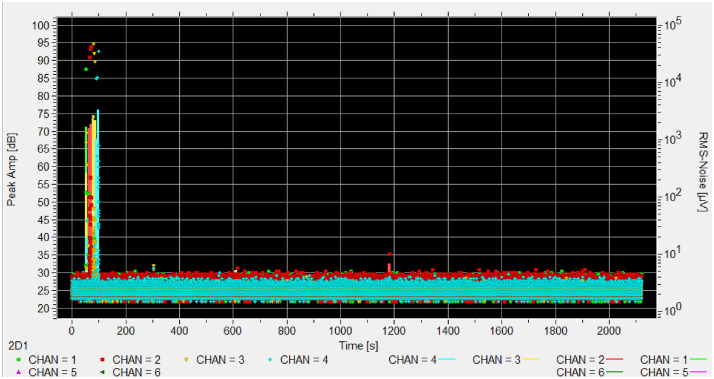
Hits amplitude in time of experiment CMH_002, no channel is infested. The peak around 80 seconds is the PLB test.

The csv archive contains computed statistics, derived from and extending the features extraction approach of Sutin and al. [11], Jiang and al. [8], and Mankin and al. [12], on the raw data as time series of sensors paired with reference. Files are computed with the script “tradb2csv.py”. Each csv file is named following this nomenclature: [experiment name]_[channel number]_[label].csv. The reference statistics are prefixed with *ref_*. The labels are 1 for infested and 0 for non-infested.

The script archive contains scripts that computed raw files or describe files. The logic and script description are on the scripts chapter of Experimental design, material and methods.

## 4 Experimental Design, Materials and Methods

### 4.1 Data acquisition

The recording of samples has been conducted by the conservation-restoration domain of the HE-Arc. The sample collection ran from April 2025 to July 2025. Test samples consist of wood from garden suspected of infestation, wood from garden without infestation, wood objects (tables, wood flooring), not infested heritage objects and infested heritage objects. As the infested were collected from outdoor environment, the species of woodworm can’t be determined. Several species might cohabitate in the same sample.

The different tests have been performed at diverse locations and different indoor ambiences. In CMH the test was done on a loading bay and in other institutions in more classic places such as conservation workshops or warehouses.

The recordings have been made using Vallen VS900-M broadband sensors (100 to 900 kHz). As the exact species of insects could not be determined, the use of broadband sensors are suitable for multi-species detection. Woodboring insects typically found in museums environment (*Anobiidae, Lyctinae [*2*]*) produce AE signals with frequency range from 25 to 500 kHz [13]. The dataset therefore may reflect mixed-species signals rather than single-species laboratory colonies.

Others equipment parts used are Vallen AEP5 Preamplifier set to 34dB gain, Vallen CBL-1-5M-V1 (RG-58) coaxial cables between preamplifier and chassis, Vallen CBL-1-1M2-V5 sensor-cables between sensor and preamplifier, Vallen AMSY-6 4 channels chassis with ASIP-2 as AE signal processor. The software used to record experiments was AE Suite R2022.0809.2.

The software was configured for continuous AE recording with a 10-second status interval and transient data acquisition using a 2 MHz sampling rate with a maximum of 1024 samples per set. Both transient recording (TR) and duration-adapted TR were enabled with data compression activated for storage optimization. Signal processing employed digital filtering with a 25 kHz to 850 kHz bandpass filter using standard configuration, while frontend filtering was disabled and notch filtering was turned off to preserve natural signal characteristics. The stimulation system operated with 1000 ms burst intervals delivering single pulses per burst at 200 Vpp amplitude with normal frequency settings and 5.2 μs AST pulse width. Timing control used 1-second parametric intervals with 10 ms clock resolution, while PCTA triggering was set at 2000 mV threshold level with -1000 mV hysteresis and record control limits established at ±10240 mV range.

Each input channel (PA0 to PA3) was configured identically with 10V input range, and all channels enabled simultaneous data collection. Both AE and TR detection were activated on each channel with a 30.1 dB detection threshold, 8192 μs duration discrimination time, and 8192 μs rearm time, while the peak detection window was set to zero for immediate response. Signal conditioning employed 34 dB preamplifier gain through AEP4/AEP5 devices, and TR acquisition used IIR5 normal data selection.

Each experiment has been set up with a sensor used as a reference to record the ambient noise. It's placed upside down near the tested object, the ceramic plate facing up. Other sensors are placed on the objects and labeled with their channels number to easily understand the picture of the set-up. The chassis is placed on another surface than the object to avoid AE interference.

We used three kinds of coupling method between sensors and object depending on the fragility, and museum restrictions, test duration and the capacity of the material to stick to the object. We employed mostly Renaissance® wax (we applied a thin layer on the sensor; we disposed the sensor on the object; if the coupling is hazardous, we secure it with ribbons or weights). Solvent-free cyclododecane spray has been used, when possible, because of its better compatibility with heritage objects [5] (we spray a thin layer of cyclododecane on the object; we quickly applied the sensor on the object and apply a second layer of CDD around the sensor; if the coupling is hazardous, we secure it with ribbons or weights). When other possibilities were not applicable, we used only weight and ribbons to dispose the sensor.

At the beginning of each test, we perform a pencil lead break (PLB) test on each sensor to verify that the setup is fully functional and to accurately identify which sensor corresponds to which channel.

The PLB consists of breaking a mechanical pencil lead three times on the surface of the tested object, at a distance of 1cm from the sensor. The mechanical pencil used is the Hsu-Nielsen Source produced by Vallen, with 0.5mm 2H lead, with a 30° breaking angle guide.

The lead break generates a large signal spike that is clearly visible on the monitor. For long-duration tests, the PLB test is repeated every 24 hours. For tests running over weekends, the PLB test is performed at the end of the test period.

### 4.2 Labels determination

Infestations labels (“infested” / “not infested”) were assigned through a three-step evaluation procedure combining

(A) acoustic emission pattern analysis;
(B) temporal and context verification;
(C) independent physical confirmation.

Whenever a test is considered infested based on these criteria, the sample goes under a free-kill protocol (two one-week freezing cycles at -18°C, with 24-hour room temperature defrosting between cycle). This protocol is used in museums to kill any stages of insects (eggs, larvae, adults).

We determine a sample to be infested according to these results:

(A) AE pattern shows a dense, cloud-like hit pattern that remains sustained throughout the test with intensity changes below the PLB level.
(B) The signal persists outside work hours and exceeds the baseline noise band recorded from healthy controls.
(C) Samples stored in plastic bag are observed to detect fresh physical evidence such as frass, new exit holes, or live larvae and adults.

When visual signs are absent, and for aditionnal evidence, we employ the freeze-kill protocol to validate our findings, as authentic insect-generated acoustic emissions cease after confirmed insect mortality. The result expected to confirm infestation is the complete cessation of the cloud-like hit pattern.

We determine a sample not infested according to these results:

(A) Acoustic emissions signals consist only of baseline noise and isolated hits (such as PLB events or sporadic mechanicals events) without any continuous “cloud” of AE activity.
(B) We identify external or setup-related noise sources by detecting hits that coincide across reference sensors or multiple samples, or when transient clusters align with known disturbances such as footsteps, sensor coupling issues, or weather events.
(C) We reinforce the not-infested classification through visual inspection of the samples that confirms the absence of frass, new exit holes, or live insects.

This complete physical-validation process was only possible at the CMH, where objects could be monitored for a longer period and frozen. The NGC data showed no detectable AE activity indicative of infestation and all samples were labelled “not infested” based on AE and environmental context. At MNA and HE-Arc, physical confirmation and freeze-kill treatment were not possible due to time and institutional constraints. For these sites, labels were assigned using the AE-based criteria described previously and the contextual information only.

### 4.3 Scripts

All python scripts required to reproduce statistical CSV files and figures are provided in the scripts archive on the Zenodo repository. All the scripts have their own configuration file, and only the data folder should be adjusted to the user's local environment.

The “tradb2csv.py” script parses the uncompressed raw archive and takes each “.tradb” file one by one. It extracts a 20-minutes chunk and computes each hit statistics per channel (18). When all the chunks are processed, each non-reference channel is paired with the references channel and exported on a CSV file. It results of three (n, 36) shape CSV files named following the convention: [experiment name]_[channel number]_[label].csv.

## Limitations

The dataset presents limitations that may affect its generalizability and application. The data collection period is restricted to four months (April to July 2025), potentially missing seasonal variation in woodboring insect activity. Geographic representation is limited to five sites across Western countries (Switzerland, Canada, France), which may not adequately represent the diversity of climates, wood species, and insect populations found globally. The use of different coupling methods (renaissance wax, cyclododecane, ribbons and weights) across experiments introduces variability in acoustic signal transmission that could confound machine learning models and affect detection consistency. Labeling reliability is limited by relying on a single acoustic emission expert for infestation determination. The binary classification system (infested/not infested) simplify real-world, omitting valuable information about infestation severity, specific insect species, developmental stages. Finally, ambient noise recorded during experiments may enable sample differentiation based on environmental conditions rather than actual insect-generated acoustic signatures.

## Ethics Statement

The authors have read and follow the ethical requirements for publication in Data in Brief. The current work does not involve human subjects, animal experiments, or any data collected from social media platforms.

## CRediT Author Statement

**Tom Marti:** scripting, data analysis, manuscript writing and review

**Cécile Costa:** design and set-up of the experiment, AE data acquisition, manuscript review

**Emmanuel De Salis:** data analysis and scripting review, manuscript review, supervision

**Laura Brambilla:** preliminary tests and acquisitions, manuscript review, supervision

**Stefano Carrino:** preliminary tests and acquisitions, manuscript review, supervision

## Data Availability

ZenodoA dataset of acoustics emissions recordings of woodboring insects on wood and cultural objects, context images and remarks. (Original data)
